# Beyond conservation agriculture

**DOI:** 10.3389/fpls.2015.00870

**Published:** 2015-10-28

**Authors:** Ken E. Giller, Jens A. Andersson, Marc Corbeels, John Kirkegaard, David Mortensen, Olaf Erenstein, Bernard Vanlauwe

**Affiliations:** ^1^Plant Production Systems, Wageningen UniversityWageningen, Netherlands; ^2^Sustainable Intensification Program, International Maize and Wheat Improvement Center (CIMMYT)Texcoco, Mexico; ^3^Agro-ecology and Sustainable Intensification of Annual Crops, French Agricultural Research Centre for International Development (CIRAD), c/o Embrapa-CerradosPlanaltina, Brazil; ^4^CSIRO Agriculture, Commonwealth Scientific and Industrial Research Organisation AgricultureCanberra, ACT, Australia; ^5^Department of Plant Science, The Pennsylvania State UniversityUniversity Park, PA, USA; ^6^Socio-economics Program, International Maize and Wheat Improvement Center (CIMMYT)Texcoco, Mexico; ^7^Natural Resource Management Research Area, International Institute of Tropical AgricultureNairobi, Kenya

**Keywords:** sustainable intensification, soil erosion, mulch, legumes, systems agronomy, climate smart agriculture

## Abstract

Global support for Conservation Agriculture (CA) as a pathway to Sustainable Intensification is strong. CA revolves around three principles: no-till (or minimal soil disturbance), soil cover, and crop rotation. The benefits arising from the ease of crop management, energy/cost/time savings, and soil and water conservation led to widespread adoption of CA, particularly on large farms in the Americas and Australia, where farmers harness the tools of modern science: highly-sophisticated machines, potent agrochemicals, and biotechnology. Over the past 10 years CA has been promoted among smallholder farmers in the (sub-) tropics, often with disappointing results. Growing evidence challenges the claims that CA increases crop yields and builds-up soil carbon although increased stability of crop yields in dry climates is evident. Our analyses suggest pragmatic adoption on larger mechanized farms, and limited uptake of CA by smallholder farmers in developing countries. We propose a rigorous, context-sensitive approach based on Systems Agronomy to analyze and explore sustainable intensification options, including the potential of CA. There is an urgent need to move beyond dogma and prescriptive approaches to provide soil and crop management options for farmers to enable the Sustainable Intensification of agriculture.

## Introduction

Food production must increase to meet the needs of a growing population whilst minimizing impacts on the environment (Foley et al., 2011). A consensus emerges that this requires the Sustainable Intensification of agriculture (Tilman et al., 2011; Garnett et al., 2013; Vanlauwe et al., 2014a). Conservation agriculture (CA) has been highlighted as a key route to Sustainable Intensification (Hobbs et al., 2008; Pretty and Bharucha, 2014).

CA is based on three principles: (1) Minimal soil disturbance or no-till; (2) Continuous soil cover—with crops, cover crops or a mulch of crop residues; (3) Crop rotation (FAO, 2015). The first two principles are inter-dependent—a mulch cannot be maintained when the soil is tilled. “True” CA is deemed to be practiced only when all three principles are meticulously applied (Derpsch et al., 2014). Yet farmers have practiced variations of the constitutive CA elements long before the term was coined.

The soil conservation imperative, triggered by the 1930s “Dust Bowl” in North America (Joel, 1937; Baveye et al., 2011) prompted the development of no-till approaches (Faulkner, 1943). The expansion of no-till agriculture in the 1980–90s in the Americas and Australia was largely driven by a combination of factors: First, effective herbicides (atrazine, paraquat, and glyphosate) were released in the 1960s and 1970s (Unger and McCalla, 1980; LeBaron et al., 2008). Second, direct seeding into a mulch of crop residues was made possible with no-till planters. The elimination of several tillage operations led to fuel savings. Third, government policy incentives supported a transition to no-till in the USA (Fuglie and Kascak, 2001). Fourth, the advent of herbicide resistant, genetically-modified (GM) crops in the mid 1990's enabled the use of highly efficacious post-emergence herbicides and accelerated the expansion of no-till and CA—particularly in the Americas (National Research Council, 2010). To different degrees, this has led to widespread adoption of no-till and CA on large farms in Australia (Llewellyn et al., 2012; Kirkegaard et al., 2014a), Brazil (Bolliger et al., 2006), and North America (Egan, 2014). By 2009 it was estimated that 62–92% of Australian farmers practiced no-till on 73–96% of their cropland (Kirkegaard et al., 2014a). By contrast, adoption by smallholder farmers is limited to only 0.3% of the farm land worldwide under CA (Derpsch et al., 2010).

The rationale for developing CA systems (i.e., reducing soil degradation and production costs), and its guiding principles and practices were considered valid for Africa and consequently sparked large interest among research organizations and funding agencies (Ekboir, 2003). The past 10 years have seen a massive wave of enthusiasm for CA among scientists, with strong support from the Food and Agriculture Organization of the United Nations (FAO). In Africa, CA is now government policy in Tanzania, Kenya, Malawi, Mozambique Zimbabwe, Zambia, and Lesotho and is actively promoted by regional organizations [e.g., the African Conservation Tillage Network (ACT), the New Partnership for Africa's Development (NEPAD), Southern African Development Community (SADC)], in research for development projects of international research centers (CIMMYT, ICRISAT, CIRAD, ICARDA, and ICRAF), by many local and international development NGOs, including many church-led organizations, and private donors such as the Howard G. Buffet Foundation.

There is a burgeoning literature on CA [including more nuanced views in recent special issues in Agriculture, Ecosystems, and Environment (Stevenson et al., 2014) and Agricultural Systems (Erenstein et al., 2015)]; numerous and diverse journal articles; two recent books (Jat et al., 2013; Farooq and Siddique, 2015) and numerous international conferences, workshops, and scientist-supported “Declarations”^1^.

The popularity of CA and the general adherence to its principles appear to be based on a number of factors. First, the belief that soil disturbance is unsustainable as it causes soil degradation/erosion and reduces soil carbon (C) stocks (Hobbs et al., 2008; Lal, 2009; Kassam et al., 2014). Second, the view that continuous no-till with crop residue retention results in “soil health” improvements which will, in time, translate to higher yields, and sustainable agriculture (Kassam et al., 2014). Failure to see yield improvements in the first 5–10 years of adoption (Rusinamhodzi et al., 2011) was therefore commonly dismissed as a transition period (Derpsch et al., 2014). Third, the name, which many interpret as meaning a form of low-external-input, biodiversity-enhancing, and sustainable agriculture. Fourth, the apparent mimicking of natural systems in which biomass remains on the soil surface and soils are not often exposed (Altieri and Nicholls, 2004). Some religious protagonists of CA thereby refer to mulch as “God's blanket” (Andersson and Giller, 2012). CA has increasingly been endorsed as Climate Smart Agriculture, contributing to both climate change adaptation, and mitigation (Harvey et al., 2013; Pretty and Bharucha, 2014).

## The many shapes of conservation agriculture across the globe

**Figure 1:**
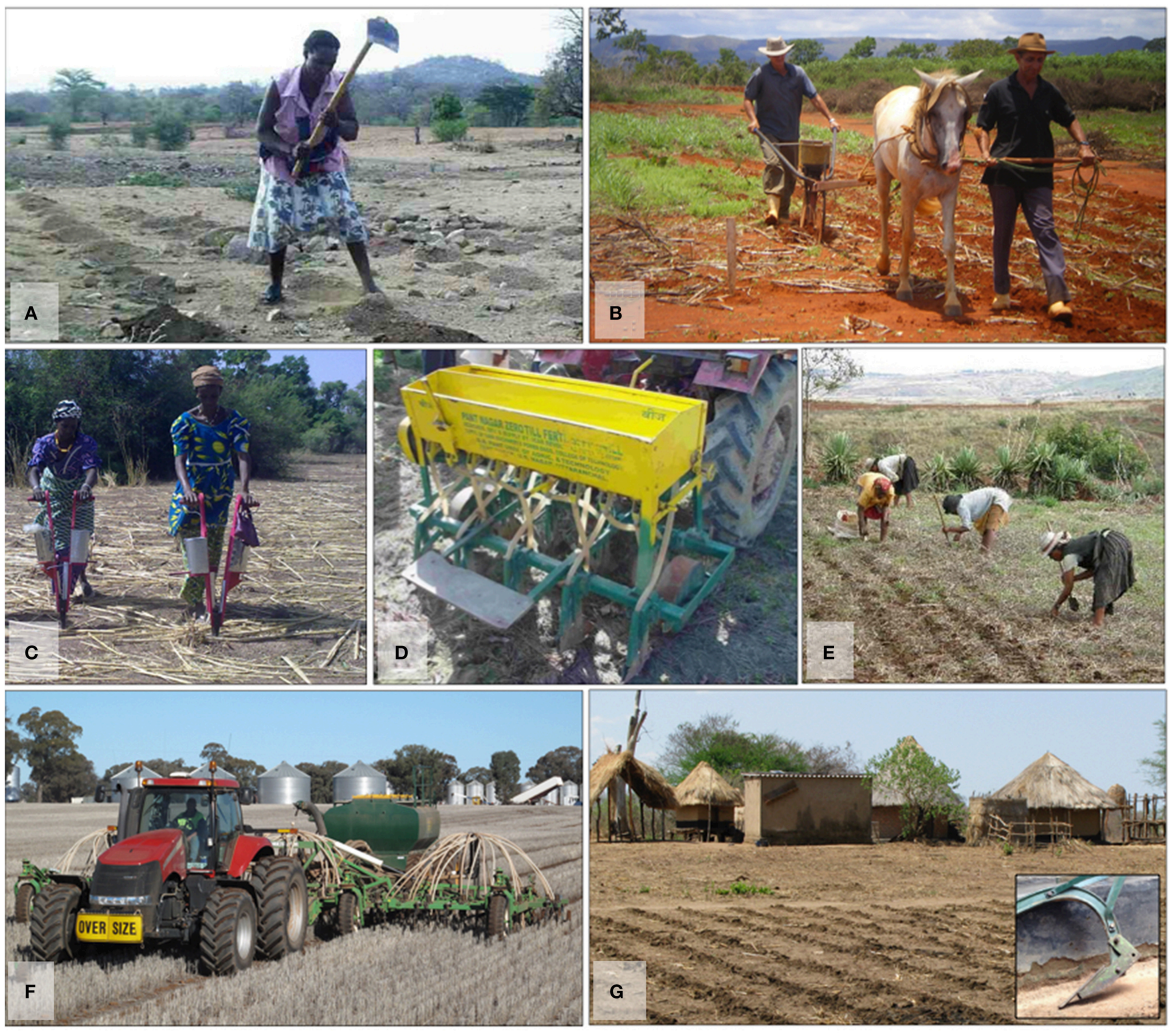
**The many forms of Conservation Agriculture across the globe. (A)** Digging planting basins using a hoe in Zimbabwe. Note the absence of crop residues. (source: Jens A. Andersson); **(B)** Seeding on no-tilled soil with a direct seeder and fertilizer distributor in Parana, southern Brazil. (source: CIRAD, France); **(C)** Direct seeding on no-tilled soil using a jab planter in Burkina Faso (source: Patrice Djamen, ACT, Kenya); **(D)** Tractor drawn zero-till seed-cum-fertilizer drill in the Indo-Gangetic Plains, India (source: Olaf Erenstein, CIMMYT, Mexico); **(E)** Direct seeding using a pointed stick in Madagascar (source: Eric Penot, CIRAD, France); **(F)** No-till, controlled traffic crop sowing in Australia using a disc-seeder with satellite guidance with 2 cm accuracy to sow between the rows of previous crop stubble. (source: CSIRO and Grass Roots Agronomy, Australia); **(G)** Field of smallholder farmer in Zimbabwe that has been minimally tilled using a Magoye ripper (see inset photo). (source: Jens A. Andersson).

Alongside the development of no-till technologies, a range of approaches and definitions emerged, such as zero-tillage, minimum tillage, conservation tillage, etc. The term “Conservation Agriculture” was coined in the late 1990s, just before the 1st World Congress on CA in Madrid in 2001, yet considerable diversity in approaches and understandings persists. While for some CA means resource conserving, low-external input agriculture, others associate it with highly industrial, glyphosate resistant, GM-based agriculture, resulting in unlikely bedfellows such as Charles, Prince of Wales (an ardent organic farmer), and the large agri-business company Monsanto. The diversity of understandings is matched by a great variety of CA practices in the world's diverse agro-ecologies and farming systems (Table 1, Figure 1).

**Table 1:** **Conservation Agriculture takes many forms across the globe related to farm size and intensity of input use**.

**Type**	**Region (examples)**	**Tillage**	**Mulch**	**Rotation**	**Farm type**	**Input**	**Main issues**	**References**
Direct planting with hand tools	Sub-humid Sub-Saharan Africa (West, East, and southern Africa)	None—use of pointed stick to plant (dibbling)	Little to no mulch of crop residues	Maize predominant—some legumes	Smallholder mixed crop-livestock farms (<3 ha)	Low level of fertilization No/limited use of herbicides	+ water conservation and erosion control if mulch present − limited biomass, CR trade-off (feed) − labor savings conditional on herbicides − rotation is limited − yield increase conditional on fertilizer	Thierfelder et al., 2014
Planting basins (conservation farming)	Semi-arid southern Africa	Localized hoeing to make planting pits		Maize/sorghum/pearl millet predominant—some legumes				Twomlow et al., 2008; Mazvimavi and Twomlow, 2009
Animal driven reduced tillage	Sub-humid southern Africa	Use of ripper or subsoiler to make planting furrows		Maize predominant—some legumes	Smallholder mixed crop-livestock farms (2–5 ha)			Thierfelder et al., 2014
Animal driven no-tillage	Subtropical southern Brazil	Use of direct seeder for planting directly through mulch into soil	Mulch of crop residues and cover crops	Maize, soybean and beans followed by winter wheat, black oats, rye, or leguminous cover crop	Medium-sized mixed crop-livestock farms (20–50 ha)	Medium level of fertilization and use of herbicides	+ control of soil erosion + longer period available for planting crops + cover crops, if inputs available − soil compaction − invading weed species	Bolliger et al., 2006
Tractor-operated no/reduced tillage (small-medium scale)	NW Indo-Gangetic Plains (India/Pakistan)	Use of no-till tractor-mounted direct seeder (locally manufactured)	Partial mulch of crop residues	Wheat crop only (in irrigated wheat-based double crop systems, e.g., rice-wheat)	Mixed crop-livestock farms (<20 ha)	Irrigation, fertilization and use of herbicides	+ reduced costs (tractor time and fuel costs) + yield effect (enhanced timeliness) − crop residue use and handling	Erenstein and Laxmi, 2008
	West Asia-North Africa (dry Mediterranean climate)		Limited mulch of crop residues	Wheat, barley, legumes (lentil, chickpea)	Mechanized mixed crop-livestock (sheep) farms (<200 ha)	Medium use of fertilizer and herbicides	+ water conservation + (wind) erosion control + increased grain yields if early sowing + fuel savings − CR trade-off (feed) − reduced seedling vigor in cereals − invading weed species	Kassam et al., 2012
Tractor operated reduced tillage (medium scale)	North-west Europe (cool temperate climate)	Some superficial soil tillage before direct seeding	Mulch of crop residues	Fodder and grain maize, wheat, barley, and cruciferous cover crops, ryegrass	Mechanized medium-scale (arable) farms (30–300 ha)	Intensive use of fertilizer and herbicides	+ control of erosion and run-off + allows earlier seeding of autumn-sown crop + fuel savings − delayed planting of spring-sown crops − topsoil compaction − increased costs with herbicides − (grass) weed problems − drainage and soil aeration problems, especially in wet season − unsuitable for incorporation of solid animal manures	Cannell, 1985; Soane et al., 2012
Tractor operated direct seeding (large scale)	Australian wheat belt (subtropical and Mediterranean climate)	Use of no-till tractor-mounted director seeder (large tractor implements)	Mulch of crop residues	Cereal-legumes (oilseed)	Mechanized large scale farms and enterprises (1000–10,000 ha)	Reliance on herbicides and fertilizer	+ reduced input costs, timeliness + erosion control, water capture − herbicide resistance − limited rotation due to greater profitability of cereals − acidification, requiring lime incorporations	Llewellyn et al., 2012; Kirkegaard et al., 2014a
	North-America (Canada and the mid-west)			Maize-soybean	Mechanized large scale farms (<500 ha)		− herbicide resistance − limited crop diversity	Hansen et al., 2015
	Cerrado region, Brazil (tropical sub-humid climate)				Mechanized large scale farms and enterprise (500–5000 ha)		+ erosion control + fuel savings + possibility to grow two crops per year − herbicide resistance − soil compaction	Bolliger et al., 2006

Objective measurement of CA adoption is challenging. None of the underlying principles is systematically captured—let alone the combination of the three principles. CA adoption figures are guesstimates—confounded by varying degrees of emphasis on one or more of the principles and interpretations (Derpsch et al., 2010). Often no-till areas are simply counted as CA adoption. Still there is increasing evidence of problems emerging with the practice and adoption of CA across the world—particularly for smallholders and less intensive systems. CA promotion in Africa and Asia often provides adoption incentives (e.g., fertilizer support) to smallholder farmers, thus creating an unwarranted policy success based on misleading yield effects and adoption figures (Andersson and D'souza, 2014; Whitfield et al., 2015).

## Emerging issues

Despite calls for a more nuanced view in the academic literature on CA's potential benefits and applicability in different agro-ecologies (see special issue of Agriculture, Ecosystems & Environment 2014 volume 187), CA continues to polarize the global R&D establishment. CA advocates, including FAO, faithfully adhere to the principles and continue to promote CA as a silver bullet that can be made to fit all circumstances (Kassam et al., 2014). Any critique or questioning of CA still provokes strong reactions from advocates—so strong that it remains impossible to discuss and debate. A common reaction to countervailing evidence—including the recent meta-analysis of Pittelkow et al. (2015a) in Nature—is that the studies have not used a correct definition of CA, that CA is a holistic approach and therefore cannot be analyzed using the tools of reductionist science (Derpsch et al., 2014). For example, questioning of the appropriateness of the widespread CA promotion across Africa in Nature (Gilbert, 2012) led to a strong retort (Buffet, 2012). The 2013 Nebraska Declaration was an attempt to seek consensus among scientists about a widening of the CA concept, but was criticized for its potential to suffocate scientific debate (Andersson et al., 2014).

Concern about the gathering momentum and funding allocation to CA among development agencies, donors and governments in Africa despite limited scientific evidence of its suitability and benefits to diverse smallholder farmers, prompted publication of a critical journal article in 2009 (Giller et al., 2009). This “heretics' view” paper provoked a storm of protest internationally^2^. Five years on, there is a growing concern about the quality of CA research and the dogmatic application of CA (Stevenson et al., 2014); both in smallholder systems and in large-scale mechanized agriculture. It is therefore timely to review the advances made and the current state of evidence.

## The evidence base for conservation agriculture

Unfortunately the wall of scientific evidence to support many of the claims made for CA is cracking at the seams—even in large-scale agriculture. In this section we analyze each of these claims in turn.

### Purported gains in yields and profitability

**Figure 2:**
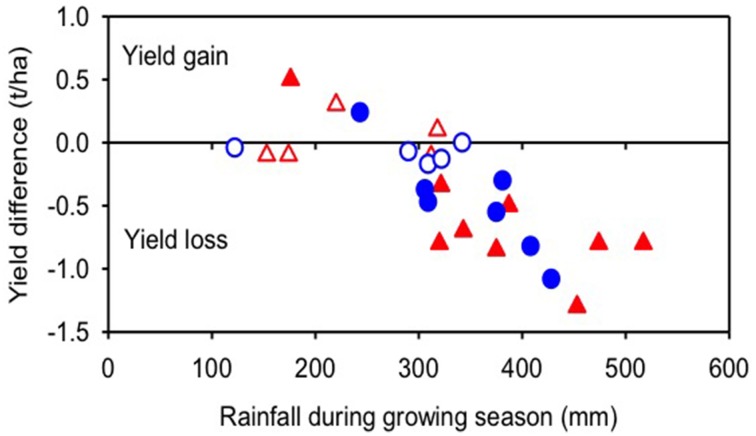
**The yield response to Conservation Agriculture also varies with seasonal conditions at individual sites**. At two long-term experiments in south-eastern Australia [Harden 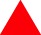 (Kirkegaard et al., 1994) and Wagga Wagga 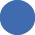 (Heenan et al., 1994)], the yield of wheat under a stubble-retain, no till (CA) treatment suffers a yield penalty compared to minimum-tillage-late burn treatment in wetter seasons (>300 mm), shows little difference in dry seasons (<250 mm) with only two instances of significantly higher yield. Open symbols are shown where treatments were not significantly different (*P* < 0.05).

Claims that CA increases crop yields do not hold up to close scrutiny. The latest comprehensive meta-analysis of 5463 paired yield observations from 610 studies suggests that no-till in itself results in a yield penalty of around 10% overall (Pittelkow et al., 2015a). Yet, this evidence also shows that a nuanced view is necessary as yield responses of crops and agro-ecologies to CA differ. The yield penalty was strongest for cereal crops: oilseeds, cotton, and legumes gave similar yields under no-till to those with tillage (Pittelkow et al., 2015b). The negative effects of no-till are minimized when combined with mulching and crop rotation in what would be considered true CA (Pittelkow et al., 2015a). Only in dry climates is an increase in crop yields observed with CA (Rusinamhodzi et al., 2011; Pittelkow et al., 2015a). Even under dryland conditions in the Middle-East (Jones, 2000; Pala et al., 2000) and in Australia there is little evidence for yield increases (Figure 2)—and certainly not increases related to lack of soil disturbance (Kirkegaard and Hunt, 2010; Kirkegaard et al., 2014a). Nitrogen (N) fertilizer was shown to compensate for the negative effects of no-till in the (sub-)tropics where lack of N is often strongly limiting, but less so in temperate climates (Lundy et al., 2015).

Yield benefits of CA under such conditions are mostly due to more timely sowing, early crop establishment, and summer fallow weed control, rather than “soil health” improvements, though soil protection and cost-savings are undisputed (Kirkegaard et al., 2014b). In the Great Plains of US and Canada, switching to no-till/stubble retention allowed replacement of fallow with opportunity cropping of oilseeds and legumes (Kirkegaard et al., 2008). In Brazil's Cerrados, land productivity benefits of CA occur through the possibility of growing two crops sequentially in the same growing season (Bolliger et al., 2006).

The enhanced timeliness of crop establishment made possible through no-till can enhance yields by better using available moisture and reducing off-season heat stress. Mulch can reduce soil temperatures, which is beneficial in heat stressed environments. By contrast, in wetter and colder climates, tillage accelerates warming, and drying of soils thereby extending the length of the growing season (Kirkegaard and Hunt, 2010). This is one of the reasons why few farmers practice no-till in northern and western Europe, whereas its use is increasing in Mediterranean countries (Soane et al., 2012).

As the capacity of CA to increase farm incomes is mainly realized through cost reductions (particularly in fuel consumption) and the capacity of large mechanized farms to “scale-up” where farming a larger area brings with it economies of scale (Egan, 2014) rather than increases in yields, the farm size, degree of mechanization, and capital investment capacity are key to understanding farmer investment in equipment and herbicides for CA. The cost reductions that can be realized with new CA technologies by—often cash-strapped—small family farms in developing countries are often very small, also because family labor is not usually monetized (Andersson and D'souza, 2014; Corbeels et al., 2014; Pannell et al., 2014).

### Soil carbon and soil fertility

The UNEP Emissions Gap Report 2013 (UNEP, 2013) repeats many earlier claims that conversion to no-till can mitigate climate change by stimulating C sequestration in the soil (Powlson et al., 2014). There is increasing acceptance that increases in soil bulk density under no-till lead to erroneous estimates of increased soil C stocks (Ellert and Bettany, 1995; Wendt and Hauser, 2013; Olson et al., 2014) and meta-analysis and literature reviews (Govaerts et al., 2009; Palm et al., 2014; Powlson et al., 2014) reveal inconsistent effects of CA. CA leads to accumulation of soil C at the surface due to the lack of soil mixing, but the impacts on increased C stocks are unclear (Powlson et al., 2014; Singh et al., 2015). There remain indications that soil C may be sequestered at depth when legumes are included in the CA crop rotation in Brazil (Boddey et al., 2010). This observation, and other evidence (Rasse et al., 2005; Kätterer et al., 2010) suggests that roots provide a substantial contribution to soil organic matter. In a global meta-analysis of the effects of CA on soil C contents (Luo et al., 2010) no-till did not increase the overall soil organic C stocks in most cases, except for those with increased biomass production and crop residue retention through growing two crops each year. The lack of an appropriate balance of the nutrients N, phosphorus (P), and sulfur (S) often limits soil organic matter build-up (Kirkby et al., 2011; Richardson et al., 2014). Part of the observed increases of soil C under CA at field level may be due to decreased soil erosion (Scopel et al., 2005).

Increased soil C accumulation in the soil surface layers, together with the presence of mulch, has important impacts on enhancing the soil water supply for crop growth. The enhanced soil structural stability and the protection of the soil surface from direct raindrop impact leads to greater infiltration and capture of rainfall for crop growth. The mulch cover can also lead to reduced soil evaporation which explains why CA shows overall yield benefits in some dry climates (Scopel et al., 2004; Pittelkow et al., 2015a). There are indications that over the long term the accumulation of soil C can lead to excess water retention and declines in crop yield (Rusinamhodzi et al., 2011). Long-term increases in soil bulk density due to the lack of soil tillage can contribute to such yield declines.

Rather than focusing solely on the use of crop residues as mulch, a more universal approach would encompass the use of fertilizers and N_2_-fixing legumes and pastures to maintain soil organic matter and crop productivity (Da Silva et al., 2014). Maintenance of soil organic matter is a key to sustainability of agricultural soils and has major benefits in mitigation and adaptation to climate change (Harvey et al., 2013). This requires N, P, and S, not just C inputs (Richardson et al., 2014).

There are few agricultural systems in the world where productivity can be sustained without import of nutrients. Nutrient import to replace nutrients removed in crops or lost requires the use of mineral fertilizer and N_2_-fixing legumes as there is insufficient organic fertilizer available globally (Connor, 2013). Mineral fertilizers can also play a critical role for the mulch component—both in terms of overcoming the problems of N-immobilization in low input systems and boosting biomass production to reduce biomass use trade-offs (Vanlauwe et al., 2014b). Even organic agriculture requires import of organic manures from other farms in the majority of systems, as well as import of nutrients as rock phosphates or feldspars. In low-external input agriculture, reliance solely on recycling is equivalent to recycling poverty (Dudal, 2002; Lal, 2007).

All approaches to maintenance of soil organic matter need to recognize the integration of livestock in mixed-farming systems. Evidence suggests little damage to soil from grazing animals when managed well (Bell et al., 2011). Efficient recycling of animal manures is a key aspect of sustainable nutrient management, both to avoid pollution and to maintain soil organic matter and crop nutrient supply. With the exception of the largely mixed farming systems in Australia (Kirkegaard et al., 2014a), crop and livestock production on large-scale farms are often separated so that competition for crop residues for mulch or livestock feeding is less important.

### Increasing diversity of cropping and integrating livestock

The benefits of crop rotation in controlling pests and disease build-up are well-established and not unique to CA (Abawi and Widmer, 2000; Kirkegaard et al., 2008). In particular, rotations with grain legumes offer the additional benefits of enhancing soil fertility through biological N_2_-fixation (Giller, 2001). Yet rotations with legumes or other crops are frequently less economically attractive and leave less soil cover (Kirkegaard et al., 2014a). Even where the profitability of cereal production is limited, smallholder farmers in developing countries often grow crops such as maize in continuous monoculture for food security reasons and their limited labor requirements (Baudron et al., 2012a).

Diverse multiple crop/pasture systems are required rather than crop rotations alone (Franzluebbers et al., 2014). This is increasingly recognized by the FAO, which has adapted their description of CA to refer to crop diversity. Whereas the adaptation of CA principles was relatively easy for dryland grains (cereals, legumes, oilseeds) it has proved much more challenging for roots and tubers, flooded rice, and cash crops like cotton and tobacco. Intercropping is particularly important on small farms in the tropics. A major benefit of multiple cropping is weed, pest and disease management.

In the USA, the overwhelming majority of maize is rotated with soybean and approximately a quarter of the maize planted is not rotated but continuously cropped for multiple years. Much of this is managed under various forms of CA. As the rotational diversity decreases, the chemical intensity associated with managing the crop increases (Davis et al., 2012). Where the economy is driven by ethanol-based biofuels (as in much of the Midwestern US) then maize is the most profitable crop. The development of the bio-fuels industry over the past 15–20 years in the US has resulted in major changes in farming practices with a much greater proportion of maize grown and a larger proportion of continuously cropped maize. In most developed economies, monoculture also is strongly driven by local economies and infrastructure investment. For example, in Nebraska, two-thirds of the maize is irrigated (USDA – National Agricultural Statistics Service, (NASS), 1970–2008). Farmers have invested in irrigation equipment and the complementary equipment to grow, cultivate, and harvest maize. Maize is the most water responsive crop in the Midwest so yields the largest return on investment.

Legume cover crops and short-duration fallows of fast-growing legume trees can fix substantial amounts of N_2_ from the air (Giller, 2001) and improve soil fertility giving strong increases in the yield of subsequent cereal crops (Sanchez, 2002), as well as providing substantial biomass for mulch (Naudin et al., 2012). Despite many claims of adoption of green manures or cover crops by smallholder farmers, these have not outlived the promotion campaigns due to the substantial investment of land and labor required and the delayed benefits to farmers (Douthwaite et al., 2002; Kiptot et al., 2007). Use of cover crops is also very limited (2% on average) on large farms in the USA (Bryant et al., 2013).

### Weed, pest, and disease management

Tillage has clear benefits in the management of biotic stresses (weeds, pests, and diseases). The repeated reliance on specific herbicides such as glyphosate has led to rapid evolution of herbicide-resistant weeds (Cerdeira et al., 2011; Mortensen et al., 2012; Kirkegaard et al., 2014a). Strategic tillage is one of the main tools that can assist in avoiding or managing such weed problems (Kirkegaard et al., 2014a). In addition to selecting for herbicide resistant weeds, the absence of tillage selects for increasing abundance of difficult to control, perennial weeds (Buhler, 1995; Smith et al., 2011). In less herbicide-intensive systems, tillage is the single most effective tactic for managing perennial weeds.

A bulky mulch can constrain crop establishment by reducing optimal seed placement, creating a suitable habitat for seed- and seedling-feeding herbivores, and impeding placement of supplemental fertilizers (Mirsky et al., 2012). Lepidopteran larvae and slugs can build up to damaging intensity in such high residue environments (Douglas et al., 2015). Residue retention and the associated increased humidity at the soil surface favor the survival of pathogens until the following crop is planted. For example, surface crop residues infected with gray leaf spot (*Cercospora zeae-maydis*) provide an early-stage inoculum for the next maize crop resulting in acute infection (Thierfelder et al., 2014). Similar problems arise with retained crop residues increasing infections of important diseases such as tan spot (*Pyrenophora tritici-repentis*) (Bhathal and Loughman, 2001) and crown rot (*Fusarium pseudograminearum*) (Burgess et al., 2001) in cereals, sclerotinia (*Sclerotinia sclerotiorum*) in legumes (Simpfendorfer et al., 2004) and blackleg (*Leptosphaeria maculans*) in *Brassica* oilseeds (West et al., 2001).

Enhanced activity of the soil macrofauna in the absence of tillage, and in particular earthworms, can alleviate excessive buildup of soil organic matter in the surface horizons (Wardle, 1995; Singh et al., 2015). The detritivore earthworm *Lumbricus terrestris* was shown to reduce the biomass of *Fusarium culmorum* in wheat straw, thus compensating for the negative effects of no-till due to crop residue accumulation (Wolfarth et al., 2011).

### Soil erosion control

A major benefit of CA is the control of soil erosion due to maintenance of soil cover, greater infiltration and reduced runoff (Roose and Barthes, 2001; Erenstein, 2002). Approximately 97% of the soil erosion reduction from adoption of no-till and CA in the US occurred prior to 1996, the year herbicide-resistant crops were first marketed, driven by price supports made possible by the US Farm Bill (USDA – Natural Resources Conservation Service, 2010). However, when no-till is practiced in the absence of effective soil mulch cover, the effects can be disastrous with rapid surface sealing leading to increased run-off and accelerated soil erosion (Guto et al., 2011; Baudron et al., 2012b). Rather than focusing on “no-till or minimal soil disturbance,” tillage, and soil conservation measures should be used strategically. Prevention of soil erosion requires a more integrated approach to soil conservation than simply no/reduced tillage and mulch. Where no-tillage is adopted, often it is practiced as non-permanent rotational tillage (Hill, 2001), and the average time out of tillage is approximately 2 years (Hill, 2001; Derpsch et al., 2010). Tillage can be important in loosening the soil and creating a rough soil surface to enhance water infiltration where mulch is not available (Aina et al., 1991). Continuous no-till can lead to soil compaction, which can be overcome by strategic tillage (USDA – Natural Resources Conservation Service, 2010; Wortmann et al., 2010).

## Trade-offs concerning conservation agriculture in smallholder agriculture

Although scientific research tends to focus on enhancement of land productivity measured in yield per unit area per season, farmers focus on maximizing productivity of all production factors (including labor and capital) with minimal risk from the whole farm over the calendar year. In particular, for farmers throughout the world their labor or time is of critical importance. Even on large-scale farms in Australia, North and South America, adoption of CA is largely driven by the ability to expand farm size, reduction in input costs, fuel, labor, timeliness of sowing, the farm program and soil protection, with less expectation that yield would necessarily improve (Llewellyn et al., 2012). In developing countries, rural households are often categorized as farmers, although they have a diverse livelihood portfolio. Many such households are net food purchasers, meaning that they spend a considerable part of their time earning money off-farm.

For smallholder, “part-time” farmers, the adherence of CA principles may imply costly or unpopular changes. One problem is the increase in labor burden, when no-till is practiced without herbicides in manual low-input systems such as in large swathes of Africa (Grabowski and Kerr, 2014). Inversion plowing is an effective means to control weeds. If herbicides are not available the labor burden for hand weeding under CA is strongly increased (Giller et al., 2009; Rusinamhodzi, 2015), limiting the CA area to what can be managed by a farming family without having to hire additional labor (Marongwe et al., 2011). The increased labor burden may be particularly strong for women. The increased drudgery of CA, particularly the form of planting basin technology (Table 1) as promoted by many church-based organizations and FAO in southern and East Africa, has led farmers to reject the technology (Andersson and Giller, 2012; Rusinamhodzi, 2015).

In addition to labor, a second problem is the competition for crop residues for soil mulching or livestock feed in smallholder farms across the (sub-) tropics that are commonly mixed crop-livestock farms. This results in CA in practice being merely no-till, with counterproductive impacts on yields, water retention and erosion control. Livestock are often key in the provision of meat and milk, of traction and manure, as well as being a means of accumulating capital and managing risk (Herrero et al., 2010). Smallholders prioritize feeding of crop residues to livestock over soil mulching (Giller et al., 2009; Naudin et al., 2014; Erenstein et al., 2015). Soil cover may be limited due to fast degradation of crop residues or removal by termites (Erenstein, 2002). Where crop productivity is poor due to exhaustion of soil fertility and soil degradation, the amounts of crop residues available are limited (Rufino et al., 2011). The need for increased productivity to produce acceptable grain yields and the crop residues needed for mulch and stockfeed suggests that use of mineral fertilizers is a pre-requisite for the success of CA (Vanlauwe et al., 2014b).

There is mounting evidence that claims for (full) CA adoption in Africa have been too optimistic as adoption is often partial (one or two principles only), limited in extent (both in terms of numbers of practicing farmers and area), and frequently temporary in nature as reports on dis-adoption suggest (Andersson and D'souza, 2014; Arslan et al., 2014). Even CA practices on small farms in Brazil, tend to be partial and on limited land areas at best (Bolliger et al., 2006). In South Asia's rice-wheat systems no-tillage is still largely confined to the wheat season (Erenstein and Laxmi, 2008). As the growing literature on CA shows, such limited and partial adoption of CA is rooted in agro-ecological and socio-economic constraints (Arslan et al., 2014), not only at the plot and farm-level, but also in the wider market, institutional and policy context (Andersson and D'souza, 2014).

## Toward a “systems agronomy”

**Figure 3:**
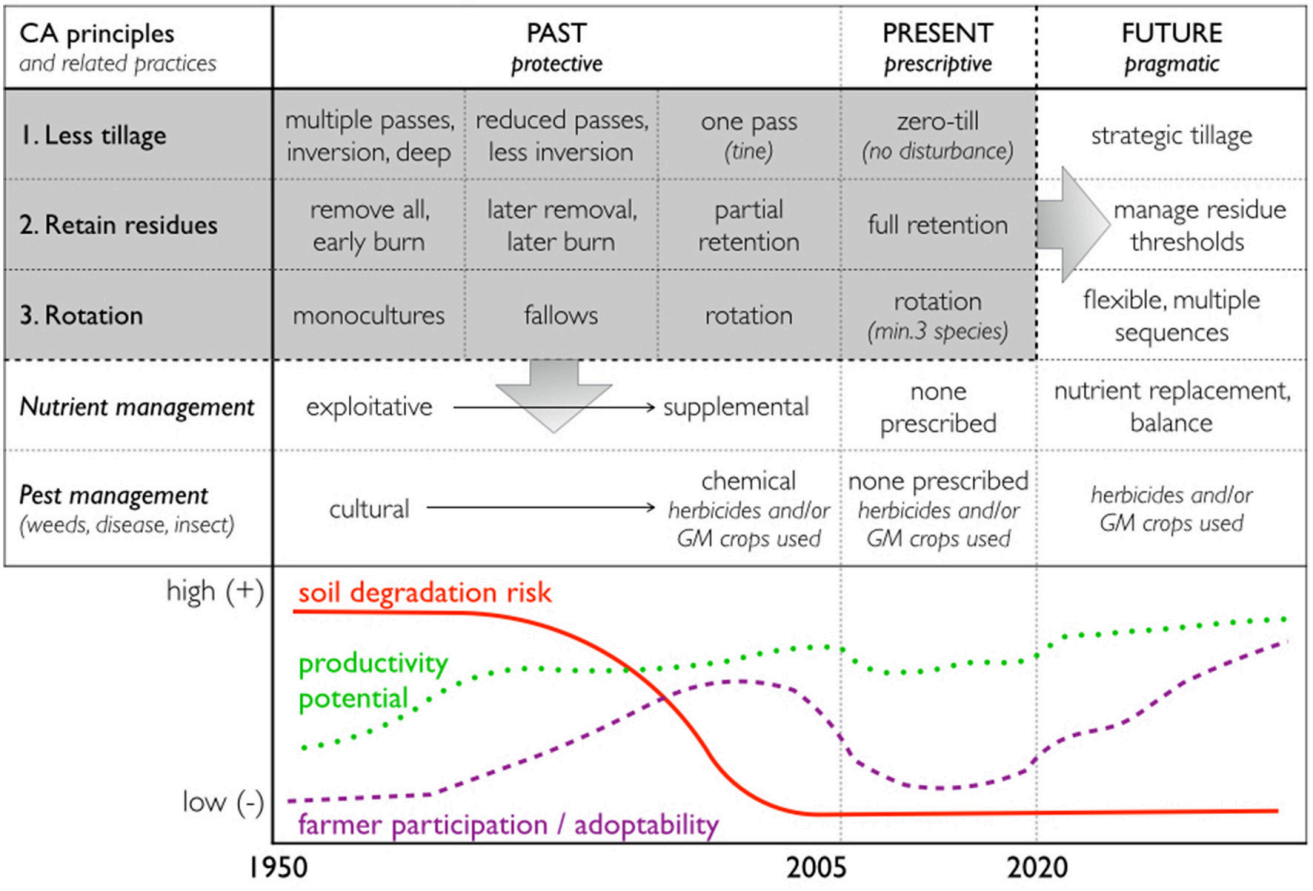
**Evolution of Conservation Agriculture practice, from PAST (conventional to no-till, Conservation Tillage), to PRESENT (Conservation Agriculture) to FUTURE (Systems Agronomy)**. There is a need to expand CA from PAST and PRESENT (i.e., the gray area) toward a Systems Agronomy (down and to the right), not losing, but adapting the three CA principles.

Our overall conclusion is that the CA principles are too narrow and restrictive to apply across the world's wide diversity of agro-ecologies and farming systems. CA places emphasis on *conservation* and thereby implicitly the status quo—in contrast to the inherent dynamism in the current drive toward sustainable *intensification*. The underlying CA *principles* also confer a value statement—norms that must be adhered to. As an alternative we suggest a *Systems Agronomy* approach, which entails a radical shift away from adapting principles or technologies to local circumstances, toward localized agronomic knowledge production (Figure 3).

**Figure 4:**
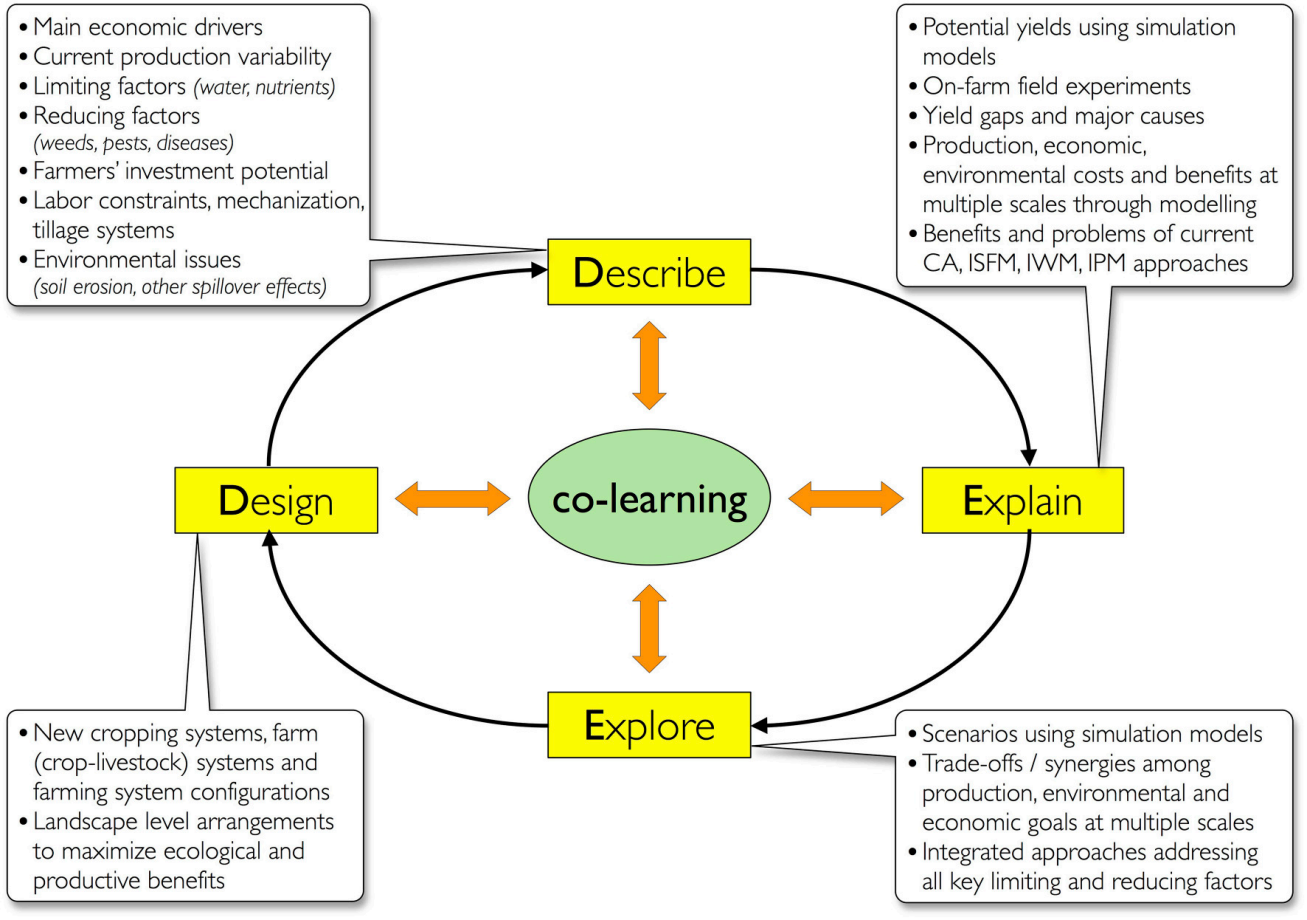
**The DEED approach (Giller et al., 2011) proposes a logical sequence of activities for researchers and farmers to learn together (Co-learning) through ***ex-ante*** analysis of the options and the prerequisite conditions (Describe), testing and analyzing options using theory, on-farm experiments and modeling to understand current practices and systems (Explain), analyzing trade-offs, opportunities and constraints to adoption of technologies at multiple scales through scenario analysis (Explore), and proposing and testing new configurations of cropping systems, farm, and farming systems and landscapes (Design)**.

Acknowledging that agronomic knowledge is fundamentally situated knowledge, the role of science is to explore systematically the needs and opportunities of the diversity of farmers in any given region. A Systems Agronomy then, is fundamentally a methodological approach which seeks to understand (describe and explain) cropping, farm household, and farming systems—nested systems—and their interactions, to explore and develop (design) a broad basket of options for diverse farming conditions and diverse contexts (Figure 4).

The theoretical foundations of a Systems Agronomy approach are the well-established principles of plant production ecology recognizing the defining, limiting and reducing factors for crop production (van Ittersum and Rabbinge, 1997). Modeling of crop growth in relation to light, water and nutrients was one of the first applications of “systems biology” (van Ittersum et al., 2003). While agronomy has tended to focus on the development of technology for crop production at the plot or field level, there is increasing recognition that the tools of systems analysis can be used at multiple levels. They can assist in identifying environmentally appropriate, economically viable, and socially acceptable technologies for farmers depending on their differing availability and access to resources, as well as their production orientations (van Ittersum et al., 2008; Giller et al., 2011; Erenstein et al., 2012).

Agronomy, and the identification and validation of new technologies or practices, thus becomes a “place-based” science in which general production ecology principles (theory) and agricultural development aspirations (direction) are applied in specific local contexts and systems. For instance, a focus on enhancing resource use efficiency (of capital, land, labor—and light, water, and nutrients), is the starting point of a sustainable intensification-directed agronomy. A Systems Agronomy perspective on Sustainable Intensification implies an empirically grounded, adaptive approach that does not merely focus on production and environment, but calls attention to social acceptability and economic viability (benefits vs. costs; private vs. public). Thus, interactions and trade-offs are taken into account between investments in different production units within a single farm and between operations on different farms within a farming system and beyond (e.g., landscape features such as refugia and buffer zones). Agronomists are thus developers of tools and providers of knowledge on farmers' realities at different scales. Such grounded knowledge can help farmers—and those organizations directly interacting with farmers—to identify and apply appropriate management options suited to their circumstances.

## Assessing current approaches to sustainable intensification from a systems agronomy perspective

Current, principle-based approaches to Sustainable Intensification, such as CA, Integrated Soil Fertility Management (ISFM), Integrated Weed Management, or Integrated Pest Management (IPM) address only specific aspects of crop management and vary in the degree to which they consider multi-scale interactions and trade-offs in farming systems. In addition, the potential of CA for intensification—yield benefits—is limited to specific agro-ecologies (Pittelkow et al., 2015a). ISFM (Vanlauwe et al., 2010) aims to increase crop productivity and maximize the efficiency of nutrient use through improved crop varieties, appropriate fertilizer, and organic inputs, all adapted to local farming conditions. Integrated Weed Management relies on multiple approaches to manage weeds that have a firmly grounded basis in ecology (Mortensen et al., 2012). IPM seeks adapted solutions to reduce or eliminate weed, insect, and disease pressure and has gone further to address multiple scales and actors, and to minimize negative environmental externalities (Brewer and Goodell, 2012).

Applying a Systems Agronomy approach to the identification of appropriate agronomic management practices to local circumstances will diffuse the emotions engulfing current debates on approaches toward Sustainable Intensification and place the ultimate beneficiaries at the center of activity where they belong.

## How can systems agronomy move science forward?

A Systems Agronomy approach goes beyond prescriptive approaches such as CA to create a “basket of options” for farmers, suited to their production conditions. Grounded analysis can inform farmers and policy makers of interactions and trade-offs (e.g., between short-term productivity increases and the longer-term sustainability) to support their technology choices. Such approaches can also learn from the ecology of natural systems and ecological theory when appropriate (Denison and McGuire, 2015).

**Figure 5:**
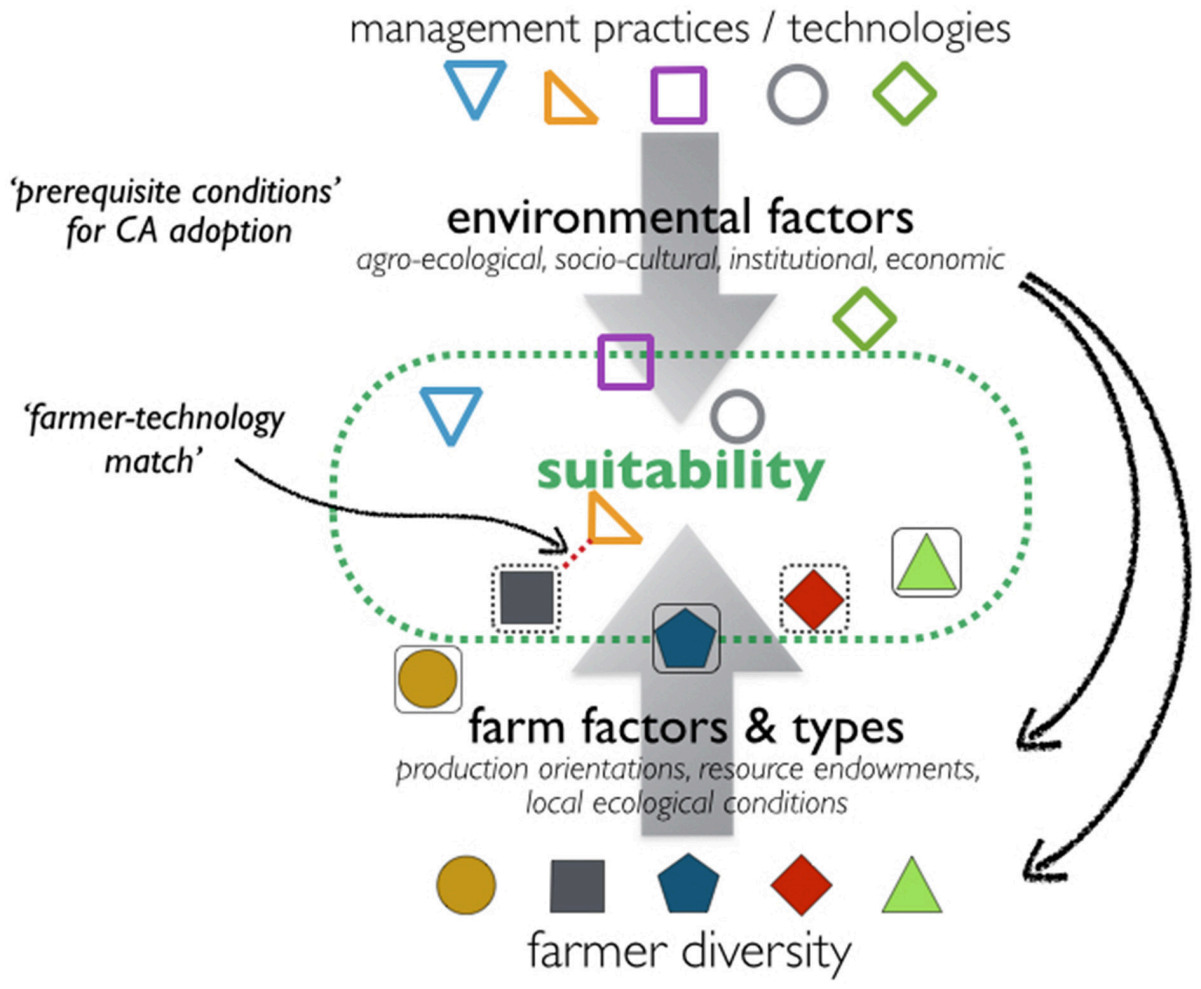
**Delineating socio-ecological niches for diverse crop/soil management practices/technologies (inspired by Sumberg, 2005; Ojiem et al., 2006)**. Using the metaphor of avoiding to force a “square peg into a round hole,” the matching of technologies to particular farmer circumstances involves (1) a selection and adaptation process of technology options suitable for the specific agro-ecological and socio-economic environment, as well as (2) a process of understanding the drivers of farmer diversity to establish for which farmers the technical options may be suitable in a given environment. Thus, we move from “Best Bet” to “Best Fit” options.

Using the metaphor of avoiding to force a “square peg into a round hole,” we depict the matching of technologies with farming systems and farmers. Different practices (technology options—sometimes called “Best Bets”), each have their specific requirements for labor, equipment, fertilizer, etc., that are suitable for different types of farmers (and farming environments) (Giller et al., 2011). In general, new, suitable technologies (or “Best Fits”) will be most rapidly adopted by smallholder farmers with adequate resources of land, cash, and labor, and not by the most resource-constrained groups (Pannell et al., 2014). Systems Agronomy entails a shift from developing “Best Bets” toward understanding “Best Fits,” grounded in farmers' realities. Farm typologies based on farmers' production orientations and resource endowments (including the importance of farm size) will help in better tailoring of technologies (Figure 5).

## Outlook and challenges

A consensus has emerged around the need for Sustainable Intensification. The focus of CA is too restricted to address the technology needs for Sustainable Intensification. It is likely that the principles underlying CA will remain a key strategy for a large proportion of farmers who have the resources to invest in mechanization, agrochemicals and herbicide-resistant crop varieties, though its implementation in practice will become more pragmatic. If current trends continue this will lead to increasing farm sizes or cooperation among large farms to justify the investment in large-scale, expensive machinery (van Vliet et al., 2015). Yet at the same time, CA will remain beyond the grasp of smallholders who lack the resources to invest in herbicides and (small-scale) mechanization or animal traction. The no-till area can be expected to increase where smallholders can access these labor-saving technologies, but with little mulching and thereby likely detrimental effects on soil and water conservation.

A more flexible approach is needed to harness the benefits of “strategic tillage” to overcome major problems associated with continuous no-till, such as soil compaction, excessive build-up of soil organic matter in the surface horizons and herbicide-resistant weeds. Herbicide use in smallholder systems also requires effective extension to avoid potential health hazards associated with incorrect use. A key role of science is to support farmers with the knowledge required to make their own strategic choices among the various appropriate technologies that are available.

A Systems Agronomy combining the tools of experimentation and simulation modeling to evaluate multi-scale trade-offs and synergies can support the development of the required knowledge. We propose that the focus should move beyond a set of narrow principles, to provide the toolbox and methods to allow informed choices of technology tailored to local conditions, and taking into account the trade-offs associated with technology choice in the short and long-term. Above all there is a need to open up debate and discussion to develop pathways for the Sustainable Intensification of agriculture.

### Conflict of interest statement

The authors declare that the research was conducted in the absence of any commercial or financial relationships that could be construed as a potential conflict of interest.

## Footnotes

^1^The 2009 New Delhi Declaration on Conservation Agriculture (http://www.fao.org/ag/ca/doc/NewDelhiDeclarationCA.pdf), The 2014 Declaration of the First Africa Congress on Conservation Agriculture (http://www.fanrpan.org/documents/d01679/), The 2013 Nebraska Declaration on Conservation Agriculture (http://www.sciencecouncil.cgiar.org/publications).

^2^https://conservationag.wordpress.com/2009/12/01/ken-gillers-paper-on-conservation-agriculture/
